# Stability and change in personal fertility ideals among US women in heterosexual relationships

**DOI:** 10.4054/demres.2018.39.16

**Published:** 2018-09-13

**Authors:** Colleen M. Ray, Sela R. Harcey, Arthur L. Greil, Stacy Tiemeyer, Julia McQuillan

**Affiliations:** 1 University of Nebraska-Lincoln, NE, USA.; 2 University of Nebraska-Lincoln, NE, USA.; 3 Alfred University, NY, USA.; 4 Oklahoma State University, OK, USA.

## Abstract

**BACKGROUND:**

Demographers typically ask about societal, not personal, fertility ideals. Societal ideals are probably more stable than personal ideals. Assessing whether personal fertility ideals are as stable as societal ideals could inform models of population fertility change and models of well-being associated with fertility outcomes.

**METHODS:**

We use the two-wave National Survey of Fertility Barriers (NSFB) to model stability and change in fertility ideals among 879 women in heterosexual couples that persisted for both waves.

**RESULTS:**

Personal fertility ideals are stable for most (69%) women, but roughly one-third adjust their ideal number between waves. Of the women who changed their personal fertility ideal, approximately half increase and half decrease their personal fertility ideal over time. Multinomial logistic regression indicates that women with a higher fertility ideal at Wave 1 had higher odds of increasing and lower odds of decreasing their fertility ideal by Wave 2. Higher education was associated with lower likelihood of increasing fertility ideals. In addition, full-time employment at the initial interview was associated with higher likelihood of decreasing fertility ideals.

**CONCLUSIONS:**

Individual characteristics, attitudes, life course, and social cues are associated with changes in personal fertility ideals. More characteristics were associated with decreases than increases in personal fertility ideals.

**CONTRIBUTIONS:**

By demonstrating that many women change personal fertility ideals over three years, the current study advances understanding of variations in fertility experiences. Importantly, these findings can also inform policies and interventions designed to support child and maternal health.

## 1. Introduction

Nearly four decades after reliable, affordable, and accessible birth control and legal abortion became available, the persistence of unrealized fertility ideals raises practical and theoretical questions about individual, cultural, social, and policy factors that contribute to individual and population level fertility (Johnson-Hanks et al. 2011). In many countries, fertility rates have declined to historically low levels (1.3 in Japan, 1.5 in Germany) (Billari and Kohler 2004; Boling 2008). The United States is unusual because it is highly developed and has maintained a somewhat high fertility rate (1.86) (Livingston 2018). Relatively low levels of fertility in developed nations have prompted some demographers to ask whether contemporary fertility behavior reflects individual preferences for few or no children, or whether there is a “fertility gap” (Chesnais 2000) that indicates lower achieved than desired fertility (Philipov et al. 2009).

Demographers tend to assume that living in a context in which people have access to contraceptive methods is associated with achieved fertility desires. Studies of fertility desires usually focus on the immediate time horizon and do not ask respondents to imagine what they would want under ideal conditions. Despite burgeoning research on fertility intentions (Johnson-Hanks et al. 2011), only recently have demographers focused on fertility ideals (i.e., ideal family size or ideal number of children) (Philipov and Bernardi 2012). Evidence from longitudinal German data that individuals sometimes revise fertility ideals (Heiland, Prskawetz, and Sanderson 2008) suggests the value of not assuming, but instead assessing, stability and change in fertility ideals. In this paper we explore two focal questions: (1) In the United States, what proportion of women in stable heterosexual unions change their personal fertility ideal over approximately three years? (2) Are individual characteristics, attitudes, life course, and social cues associated with changes in personal fertility ideals?

In the current study, we measure fertility ideals by using a question about personal ideal number of children regardless of the number of children individuals have now or intend in the future. We answer the questions above by examining a subset of women in heterosexual unions who participated in both waves of the NSFB (2004 to 2006 and 2007 to 2010), a representative sample of women ages 25–45 in the United States. We find that personal fertility ideals are stable for most (69%) women but that roughly one-third adjust their ideal number between waves. Of the women who changed their fertility ideals, approximately half increase and half decrease their fertility ideal over time. Multinomial logistic regression of the odds of an increasing or decreasing fertility ideal over time indicates that individual characteristics, attitudes, life course indicators, and social cues are associated with changes in fertility ideals.

## 2. Theoretical and empirical background

### 2.1 Intentions, ideals, and desires

Philipov and Bernardi (2012) have used the term “hypothetical fertility” to refer to a wide range of related concepts, including fertility ideals, fertility intentions, fertility desires, fertility expectations, and fertility aspirations. What all these concepts have in common is a concern with fertility outcomes that have not yet occurred and may never occur. Because fertility behavior usually involves a certain degree of planning, some demographers have argued that fertility intentions, ideals, desires, etc. are an important component of any theoretical model of human fertility (Coale 1973; van de Walle 1992). What these terms actually mean and how to distinguish one from another, however, is not always clear. Fertility intentions are usually understood to be proximate causes of fertility behavior (Ajzen and Klobas 2013; Trent 1980), and desires, preferences, and ideals are generally conceptualized as more distal (Hin et al. 2011; Miller 1994, 2011; Miller, Severy, and Pasta 2004). Fertility expectations and fertility aspirations have been used as more generic terms that can refer to fertility ideals and/or fertility intentions (Hayford 2009).

Because of persistent and fairly high rates of unintended pregnancies in the United States, there has been considerable attention to understanding fertility intentions (e.g., Mumford et al. 2016). Personal fertility ideals, although related to fertility intentions, capture unique dimensions of fertility such as early life goals and later life adjustments to realized parity. Fertility intentions are usually conceptualized as concrete plans to have a child that take into account the perceived costs and benefits of having a child at a certain point in time (Trent 1980). A great deal of evidence suggests that fertility intentions are best understood – not as relatively stable traits – but as “moving targets” (Lee 1980; Quesnel-Vallée and Morgan 2003) that can change rapidly and dramatically depending on social context. Although fertility intentions do not provide consistent predictions of achieved fertility at either the individual or aggregate level (Morgan and King 2001; Quesnel-Vallée and Morgan 2003), intentions have strong associations with the odds of giving birth (Schoen et al. 1999; Barber 2001). Although inaccuracies in predicting fertility outcomes from fertility intentions have led to questions about the utility of fertility intentions (Miller and Pasta 1995; Bongaarts 2001), the lack of a perfect correspondence does not mean that fertility intentions cannot be useful predictors. Rather, the absence of perfect correspondence provides insights into various constraints on meeting fertility intentions as well as potential problems with the measurement of the intentions construct itself (Mumford et al. 2016; Voas 2003).

Questions about fertility intentions remain common in demographic surveys, but questions about fertility ideals are less common because of perceived problems with the concept (Philipov and Bernardi 2012). The original question on ideal family size used in a 1936 Gallop Poll was “What do you think is the ideal number of children for the average American family?” (Hin et al. 2011: 135). Ryder and Westoff (1971) harshly criticized this question as lacking face validity. Without a specific point of reference, respondents do not have guidance about the intention of the question, leaving it unclear what the concept measures (Blake 1966). Philipov and Bernardi (2012), however, argue that the concept “fertility ideals” is useful even if the specific measure originally employed was not. Measures of fertility ideals can provide insights about prevailing social norms (Basten and Gu 2013; Bacci 2001; De Santis and Bacci 2001). Such measures can also inform tests of theories of microlevel fertility decision-making (Ajzen and Fishbein 1980; Basten and Gu 2013; Goldstein, Lutz, and Testa 2003; Rindfuss, Morgan, and Swicegood 1988; Schoen et al. 1999) and aid in the evaluation of theories of fertility decline (Goldstein, Lutz, and Testa 2003; Lesthaeghe and Surkyn 1988; van de Kaa 2001). In addition, factors that influence childbearing ideals may also be associated with actual fertility (Kohler 2001; van de Kaa 2001).

Measures of the ideal number of children that specify a referent may use a lens of a societal ideal family size (Goldstein, Lutz, and Testa 2003; Bacci 2001; De Santis and Bacci 2001). Thus, the Eurobarometer surveys often include the following question: “Generally speaking, what do you think is the ideal number of children for a family to have?” When the question is asked in this generic way, the ideal number of children is assumed to reflect societal norms rather than personal preferences or, as Trent (1980) puts it, the degree of pronatalism in a society. Understood as a reflection of social norms, demographers assume that the ideal number of children is relatively stable (Goldstein, Lutz, and Testa 2003; Testa and Grilli 2006). In the United States in recent decades, the two-child ideal norm (Hagewen and Morgan 2005) contributes to pressure to conform to this ideal (Morgan and Rackin 2010).

It is unclear, however, whether individual women consider the societal norm as applicable to themselves. Because theories of fertility decision-making as well as policy recommendations often focus on the role of individuals in creating macrolevel change, it is important to also measure personal fertility ideals; for example, the Eurobarometer surveys ask, “And for you personally, what would be the ideal number of children you would like to have or would have liked to have had?” Such a personal ideal number of children measure is related to fertility desires and is likely to change over time with changes in attitudes, cognitions, and social circumstances (Philipov and Bernardi 2012). In the current study, we use social psychological theories of fertility decision-making (emphasizing desires and intentions) to guide our analyses of personal fertility ideals.

### 2.2 Theoretical perspectives on personal fertility ideals

There are three major theoretical models of the process of fertility decision-making: the Traits-Desires-Intentions-Behavior (TDIB) (Miller 1994, 2011), the Theory of Planned Behavior (TPB) (Ajzen and Klobas 2013; Dommermuth, Klobas, and Lappegård 2011), and the Cognitive-Social Model (CSM) (Bachrach and Morgan 2013; Johnson-Hanks et al. 2011; Rackin and Bachrach 2016). These three compatible models have different emphases. The TDIB model was developed by Miller and his associates in 1994 and has benefited from several revisions and refinements over the past two decades (Miller 1994, 2011; Miller and Pasta 1995; Miller, Rodgers, and Pasta 2010; Miller, Severy, and Pasta 2004). The basic model posits that,

…the motivational forces driving the fertility related behaviors of individuals and couples unfold in a sequential process that begins with nonconscious motivational dispositions (traits) to have or not have children, which lead to conscious desires to have children or not, which in turn lead to conscious intentions to have children or not, which finally lead to the performance of behaviors that are instrumental in the achievement or avoidance of the childbearing

(Miller 2011: 76).

Miller’s model focuses on childbearing in a way that implies the relevance of, but does not explicitly measure, overall fertility ideals. In the TDIB model, desires constitute a conscious wish to have children and mediate between an inchoate trait that motivates desiring children or not and behaviors and conscious intentions to have children or not. The TDIB model pays more attention than other theories to underlying genetically based motivations to have or not have children (Basten and Gu 2013).

The TPB represents a general approach explaining intentional and reasoned action (Ajzen 1991, 2005), which has been applied to reproductive decision-making (Ajzen and Klobas 2013; Dommermuth, Klobas, and Lappegård 2011). TPB conceptualizes having a child as the result of fertility decision-making based upon intentions and actual control over having a child. Fertility intentions derive from three factors: attitude toward having a child, subjective norm for having a child, and perceived control over having a child. The three factors are shaped by relevant behavioral beliefs about the costs and benefits of having a child, beliefs about social support for having a child, and beliefs about factors that might enhance or restrain ability to have a child. Finally, behavioral beliefs are shaped by individual, demographic, and societal background factors. In the TPB model, personal fertility ideals are probably unstable and are values that are part of individual background factors (Philipov and Bernardi 2012). Thus. in the TPB model, the association of a personal ideal number of children with fertility intentions is explained by attitudes and behavioral beliefs. The TPB model pays more attention to underlying cognitive structures of fertility decisions than other theories.

The CSM was developed to address concerns about the overemphasis of the TPB on intentional action and proximate causes of fertility behavior (Bachrach and Morgan 2013; Johnson-Hanks et al. 2011; Rackin and Bachrach 2016). Based on recent ideas in cognitive science and social science more generally, the CSM posits that fertility behavior is the result both of conscious and deliberative intentions and nondeliberative, emotionally laden, “automatic” cognitions. The CSM posits that cognitions derive from social structures, which include both material structures and schemas (relatively stable and abstract representations of the meaning of an object or event) (Bachrach 2014). Schemas are shaped by background factors, experience, and life-course transitions. Schemas can change with life experience, such as friends or families having a baby, increasing or decreasing religiosity, changing job status, changes in media portrayals of normative family size, or changes in social policy. Of the three models, the CSM is both less specific about the elements that go into fertility decision-making and more faithful to the actual ways people think and act (Ní Bhrolcháin and Beaujouan 2015; Philipov and Bernardi 2012). In the CSM, fertility ideals would probably fit into the category of schemas insofar as they comprise representations of the meanings of fertility. More than other models, the CSM draws our attention to such likely sources of change in fertility ideals as changes in attitudes and resources, personal experiences, and life course transitions. For this reason, the CSM seems better suited for guiding research on sources of change and stability in fertility ideals than TDIB and TPB.

### 2.3 Values, resources, attitudes, and fertility ideals

Why might fertility ideals vary among women? Hayford (2009) argues that societal schemas about the ideal number of children will have the strongest association with fertility ideals. Even in a society with a strong two-child norm, there is evidence that fertility ideal schemas differ between women (Rackin and Bachrach 2016). Heiland, Prskawetz, and Sanderson (2008) discuss three sources of between-person differences in fertility ideals: family background, individual characteristics, and behavioral dispositions. Growing up in a larger family is associated with a higher ideal number of children than growing up in a smaller family (Axinn, Clarkberg, and Thornton 1994; Dey and Wasoff 2010; Gustavus and Nam 1970; Heiland, Prskawetz, and Sanderson 2008; Johnson and Freymeyer 1989). Therefore, family-of-origin experiences can shape schemas of family size (Basten and Gu 2013; Testa and Grilli 2006). Community fertility rates can also influence schemas of ideal family size (Goldstein, Lutz, and Testa 2003; Kohler 2001; Testa and Grilli 2006). Having parents who divorced is also associated with lower fertility ideals (Axinn and Thornton 1996). We therefore recognize that early life experiences can shape adult fertility ideals, but we do not have measures to directly assess these variables in the study dataset.

Stable individual characteristics are also associated with variation in fertility ideals. In the United States, there is some evidence that African Americans have lower fertility expectations than do non-Hispanic whites (Rackin and Bachrach 2016). Not all studies yield consistent results, yet most evidence, primarily from Europe, suggests that higher levels of education are associated with higher fertility ideals (Dey and Wasoff 2010; Heiland, Prskawetz, and Sanderson 2005; Hin et al. 2011; Miller 1992; Testa and Grilli 2006). Some of the inconsistency in the association of education and fertility ideals could reflect differences between lower and higher fertility contexts (Johnson-Hanks et al 2011). Higher levels of education are associated with higher fertility ideals, yet there is some evidence that being in a more prestigious occupation is associated with lower fertility ideals (Dey and Wasoff 2010). Among women, anticipated labor force participation is associated with lower fertility ideals (Waite and Stolzenberg 1976). The effect of economic resources and economic hardship on fertility ideals is not clear; higher incomes may raise ideals (Miller 1994), but an increased emphasis on quality of offspring may lower fertility ideals. In a Scottish study, Dey and Wasoff (2010) found higher income to be associated with lower fertility ideals. Social policies that reduce conflicts between work and childrearing probably influence fertility ideals for women (Mills et al. 2011).

A number of studies find that attitudes, values, and behavioral dispositions are associated with fertility ideals (Hin et al. 2011). The idea that women who place a high value on leisure or career success have lower fertility preferences is implied in some theories of fertility (e.g., Bongaarts 2001) and explicit in others (e.g., Bachrach and Morgan 2011). Preference theory (Hakim, 2003, 2004) and other ideology-based theories (e.g., Lesthaeghe and van de Kaa 1986) are explicit in stressing the importance of values for fertility intentions. Hakim (2003) suggests that, of the three groups of women she identifies (work-focused, home-focused, or combined focus), the work-focused are likely to have the lowest fertility ideals.

Religiosity is also associated with fertility preferences (Koropeckyj-Cox and Pendell 2007; Pearce 2002). Religious attendance (Adserà 2006; Rackin and Bachrach 2016) and religious affiliation (Adserà 2006; Heiland, Prskawetz, and Sanderson 2008) are both associated with higher fertility desires. Using European Fertility and Family Surveys, Philipov and Berghammer (2007) found that higher scores on all religiosity measures were associated with higher fertility ideals. The effect of religiosity was much greater for fertility ideals than for fertility intentions. Philipov and Berghammer (2007) suggested that the effects of religion on fertility ideals were probably due to religious socialization, norms in religious institutions, higher levels of social capital, and the reduction of uncertainty that comes with religious beliefs.

It seems reasonable to expect that changes in fertility intentions will be associated with changes in fertility ideals. Heiland, Prskawetz, and Sanderson (2008) discovered that women with the normative ideal of two children were less likely to change their fertility ideals than women with higher or lower fertility ideals. By including a measure of fertility intentions in our model, we assess whether intentions and ideals merely capture the same underlying processes (i.e., have very high associations) or if they capture unique dimensions of fertility experiences. Prior research shows that higher importance of motherhood is associated with higher intentions (McQuillan et al. 2015), and therefore is likely to also be associated with higher fertility ideals. Therefore, we include measures of initial intentions and change in importance of motherhood.

### 2.4 Sources of change in fertility ideals

Life course theory and empirical evidence suggest that life course and social cues such as getting married, number of children, or reaching a certain age should also be associated with changes in fertility preferences (Namboodiri 1983). Ní Bhrolcháin and Beaujouan’s (2015) Preference Construction Theory holds that people do not actually have fertility preferences; rather, when they are asked questions about preferences, they construct their preferences based on current schemas and contemporary circumstances. Therefore, Preference Construction Theory suggests that fertility ideals are more like states than stable traits and are susceptible to change.

There is evidence that fertility preferences are shaped in contexts of social networks and couple relationships (Becker 1999; DeRose, Dodoo, and Patil 2002; Miller, Severy, and Pasta 2004; Thomson 1997; Voas 2003). Therefore, the importance of children to partners, the importance of children to parents, and having friends have children are all likely to be associated with fertility ideals. A higher current number of children (parity) is also likely to be associated with higher fertility ideals (Engelhardt 2004; Heiland, Prskawetz, and Sanderson 2008). Dey and Wasoff (2010) found in a Scottish sample that those with a fertility ideal of zero were more likely to have no children. There is evidence that age has a negative association with fertility ideals; higher ideals among younger people tend to adjust downward with age (Dey and Wasoff 2010; Liefbroer 2008).

There is some evidence that religion may be associated with changes in fertility ideal. One study found that Catholic women are more likely to change fertility desires than women from other religious groups (Heiland, Prskawetz, and Sanderson 2008). Employment is associated with lower fertility desires (Engelhardt 2004), and there is also evidence that a spell of unemployment is associated with a decrease in fertility ideals (Heiland, Prskawetz, and Sanderson 2008). Testa and Grilli (2006) found in a European study that being enrolled in school was associated with lower fertility ideals for women but higher fertility ideals for men.

### 2.5 Statement of problem

In this paper we analyze the initial level of and change in personal fertility ideals among US women over the course of three years. Rather than assume that fertility ideals simply mirror cultural norms in a society and are a stable trait (Naboodiri 1983), we estimate the initial distribution and change in personal fertility ideals over three years. We estimate associations of individual characteristics, attitudes, and life course cues with initial fertility ideals and change in those ideals. We focus on five sets of expectations based upon prior research and the CSM. First, women who have backgrounds associated with higher fertility rates (religiosity, friends with children) will have higher fertility ideals, and increases in religiosity or friends with children will be associated with increases in fertility ideals. Second, women who perceive that parents or partners want children will have higher fertility ideals or will be less likely to decrease fertility ideals. Third, women with higher education, full-time employment, and higher importance of leisure and work will be more likely to decrease their personal fertility ideals. Fourth, increases in parity or the importance of motherhood will be associated with higher likelihood of increases in fertility ideals. Finally, more economic hardship will be associated with a decrease in fertility ideals.

## 3. Data and methods

We use both waves of the NSFB (Johnson et al. 2009), a random-digit-dialing (RDD) telephone survey of 4,712 women of childbearing ages (25 to 45) in Wave 1. Initial interviews (Wave 1) were conducted between 2004 and 2006. Follow-up interviews were conducted approximately three years later with all women who could be reached between 2008 and 2010 (Wave 2). Using Census central office codes, high minority population areas were oversampled; therefore, we use weighted analyses. Internal review board approval was obtained from the institutions collecting the data. Information about the data, and access to the data files for public access can be found through the Inter-university Consortium for Political and Social Research (ICPSR) (Johnson 2010).

The estimated response rate for the screener sample is 53%; the response rate for the completed sample is 37%. Despite relatively low response rates, this sample is similar to other RDD telephone surveys of the same time period and is mostly representative of the population based on comparisons with Census data. There is some overrepresentation of more highly educated women. Wave 2 contains 2,136 women participants, which is 58% of those that participated in Wave 1. Only 158 (6%) of women refused to participate in the second interview; others were difficult to recontact because of the rapid increase in cell-only households and mobility due to the economic crisis of 2007. The analytic sample consists of women (N = 879) who were interviewed at both Wave 1 and Wave 2, who reported fertility ideals, and who were part of the same couple at both time points.

### 3.1 Concepts and measures

The dependent variable is personal fertility ideal. The question is identical to the question asked in 1995 in Cycle 5 of the National Survey of Fertility Growth: “The next question asks about the number of children you consider ideal for yourself. This could be more or less than you already have or more or less than you expect to have. If you yourself could choose exactly the number of children to have in your whole life, how many would you choose?” Participants provided a number from 0 to as high as they wanted. This question comes near the beginning of the survey (question 12 of about 100 questions) and after questions about the participant’s pregnancy history, desires, and intentions. Question 10 is about desire for a child (“Would you, yourself, like to have a baby?”), and if relevant, about one’s partner’s desire for a child (question 10b). The next questions are about fertility intentions. (Question 11: “I’ve asked what you would like to do. Now, I want to ask about what you actually intend to do. Do you intend to have a baby?”) We did not include both desires and intentions because they are highly correlated (r = .770) and we wanted to avoid multicollinearity. Fertility ideals, however, have modest correlations with fertility desires (r = .238) and fertility intentions (r = .122). Schwarz and Strack (1990) find that survey questions on related concepts that are asked close together in a survey are easier for participants to differentiate than questions that are not close together, supporting our argument that fertility ideals may be considered as distinct from desires and intentions. The response for personal fertility ideals at Wave 1 is also included in the analyses. To help with floor and ceiling effects, the variable has been collapsed into three categories (0–1, 2, and 3 or more), with a personal ideal number of children of two serving as the reference category.

#### Individual characteristics.

Education is a continuous variable that measures the years of education from 4–22. Following Johnson (2005) and Su (2012), all of the variables that change over time have two measures: the Wave 1 measure and the change score (Wave 2–Wave 1) version. Those employed over 35 hours a week were coded as employed full time, and those working less than 35 hours a week were coded as employed part time; both groups are compared to the unemployed. We assigned a value of “3” to employed full time, a value of “2” to employed part time, and a value of “1” to unemployed. To create our measure of employment status change, we subtracted Wave 1 from Wave 2. Economic hardship is a scale computed by calculating the mean of the following questions: (1) “During the last 12 months, how often did it happen that you had trouble paying bills?” (2) “During the last 12 months, how often did it happen that you did not have enough money to buy food, clothes, or other things your household needed?” (3) “During the last 12 months, how often did it happen that you did not have enough money to pay for medical care?” Responses range from never to very often (coded so that higher values indicate more hardship). The scale is unidimensional with high reliability (α = .82).

#### Attitudes.

Importance of leisure was measured by the question “How important is having leisure to enjoy your own interests?” Responses range from not important (1) to very important (4). Importance of valuing career was measured by the question “How important is being successful in your line of work?” Religiosity was measured by four questions: (1) “How often do you attend religious services?” (2) “About how often do you pray?” (3) “How close do you feel to God most of the time?” (4) “In general, how much would you say your religious beliefs influence your daily life?” The measures were coded so that high values indicate higher religiosity, standardized, and combined into a scale by computing the mean to create a single factor scale (α = .78). Fertility intentions were measured by two questions: (1) “Do you intend to have a baby?” (2) “Of course, sometimes things do not work out exactly as we intend them to, or something makes us change our minds. In your case, how sure are you that you will/will not have a child?” Responses were coded so that low scores indicate “very sure do not intend” (−2) to high scores of “very sure do intend” (+2). Women who said they “don’t know” their intentions, who said they cannot have children, or who said they would let God or nature decide are coded 0 (the center of the scale). Four of the importance of motherhood items have responses ranging from strongly agree to strongly disagree: (1) “Having children is important to my feeling complete as a woman.” (2) “I always thought I would be a parent.” (3) “I think my life will be or is more fulfilling with children.” (4) “It is important for me to have children.” The fifth variable, (5) “How important is each of the following in your life…raising children?” has responses ranging from not very important to very important. The measures were coded so that high values indicate higher importance of motherhood and were combined into a scale by computing the mean to create a single factor scale (α = .86).

#### Life course and social cues.

Importance to parents is measured by the statement “It is important to my parents that I have children” (4 = very important). Importance to partner was measured by the statement “It is important to my partner that we have children” (4 = very important). Children amongst friends and family was measured by the question “Thinking about your family and friends, would you say that all, most, some, few, or none of them have kids?” Changes in parity were computed from a reproductive history that measured the number of live births and recoded into a series of dummy variables for always no children, always one child, always three or more children, became a parent, and added a child. Two children at both waves is the reference category. Age was measured in years.

### 3.2 Analytic strategy

To assess within-person change in personal fertility ideals, it is common to use a change score (Wave 1 scores subtracted from Wave 2 scores) as the dependent variable in an ordinary least squares (OLS) regression (Allison 1990). One shortcoming of an OLS approach is that it focuses on means and is therefore unable to detect change when some individuals increase while others decrease. In such situations, relying on means leads to the erroneous conclusion that change has not occurred, when in fact people are changing, but in opposing directions. For this reason, we recoded change scores into three categories – decreased, stable, or increased – in personal ideal number of children. Then we used multinomial logistic regression to assess associations between increases or decreases in fertility ideals, on the one hand, and stability on the other. Using conditional change scores as independent variables can lead to biased results because of the failure to consider the correlation between the independent variable measured at Wave 1 and the corresponding change score variable (Finkel 1995). Therefore, we included initial and change score versions of the independent variables. We also include indicator variables for initial low (0,1) or high (3,3+) compared to the normative (2) personal fertility ideal to assess whether the initial personal fertility ideal is associated with the likelihood of increasing, decreasing, or remaining stable. As with fixed effects and change score models, our multinomial logistic regression includes two waves of data and thus reduces concern about measurement error and omitted variables (Johnson 2005; Su 2012).

**Figure 1:**
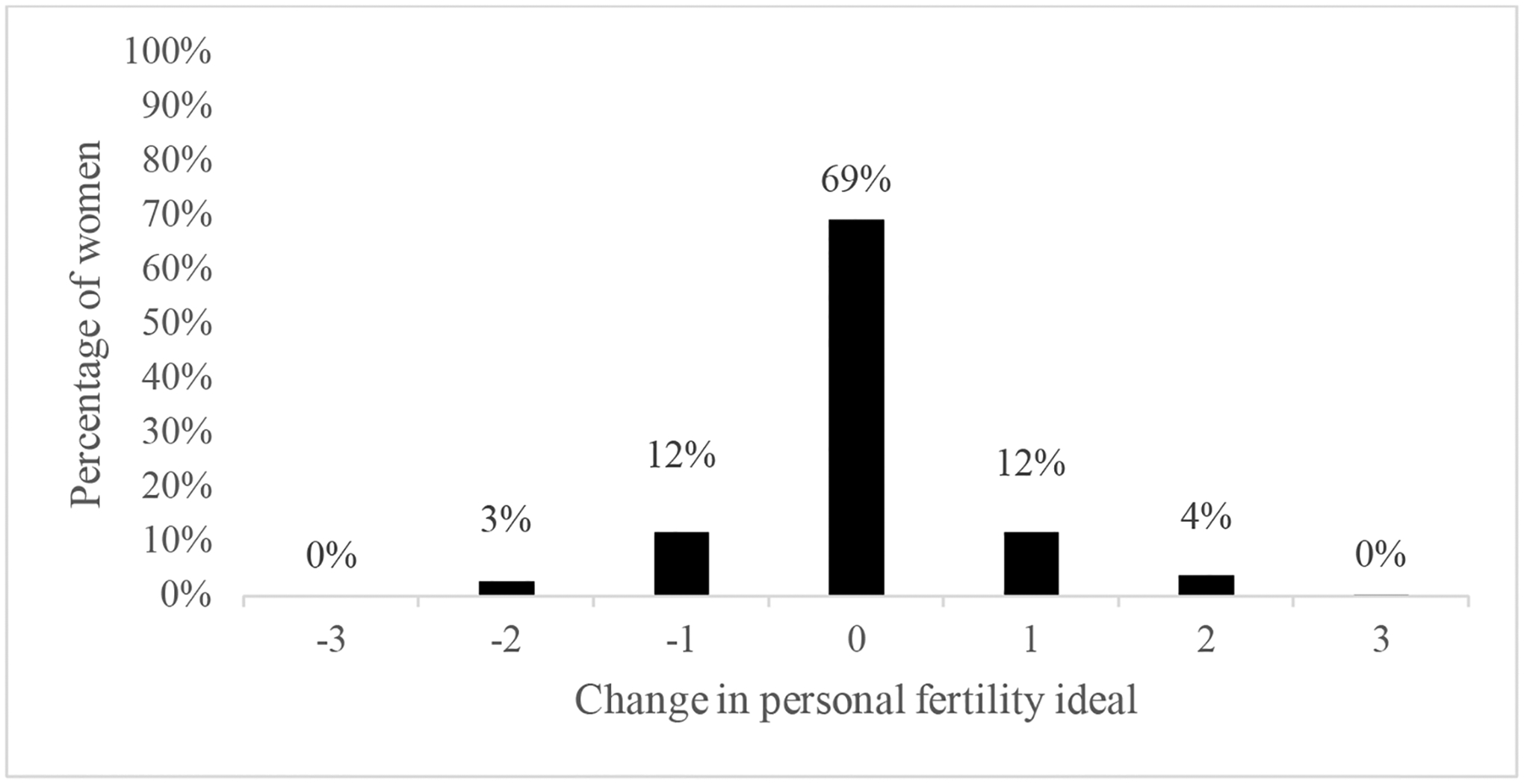
Change in personal fertility ideal between waves

A frequency distribution of the change score for personal fertility ideals indicated that 31% changed their personal fertility ideals. Figure 1 shows that, among the women who change their personal ideal number of children between waves, about half reported a higher personal ideal number of children at Wave 2, while the other half reported a lower personal ideal number of children. As noted above, we therefore created a three-category dependent variable (decreased, stable, or increased) and used multinomial logistic regression to estimate associations of individual characteristics, attitudes, life course, and social cues with decreases or increases compared to stable fertility ideals.

## 4. Results

Table 1 provides descriptive statistics for the variables in the model. Even with 31% of women changing fertility ideals, the mean is very similar across waves (2.59 for Wave 1 and 2.60 in Wave 2), in part because, of those who change, half increase and half decrease. The analytic sample consists of women in the same heterosexual relationship who participated in both waves. The mean level of education (15.80) was high, most of the women were employed full time, and economic hardship was low, with little change.

Most women had high and stable importance of leisure and career. There was little average change in religiosity between waves. Fertility intentions were initially low and on average decreased between waves. Average importance of motherhood was above the midpoint of the scale, with little average change between waves. Similarly, mean scores for the perceptions of partners’ and parents’ desires were high, as were the number of friends with children, indicating mostly pronatalist inclinations and influences. About 20% of the women did not have children, and nearly 40% had two or more children at both waves. More than a fourth had a child between waves; for about one-third of this group, this was their first child.

Table 2 provides the results of multinomial logistic regressions showing associations with decreases and increases compared to stability in personal fertility ideals. The first set of columns in the table shows relative risk ratios for the odds of decreasing personal fertility ideals, compared to stability. Values of less than one indicate decreased odds of decreasing fertility ideals and values more than one indicated increased odds of decreasing personal fertility ideals relative to remaining stable. The second set of columns shows the odds of increasing personal fertility ideals relative to stability.

As we expected, higher initial fertility ideals were associated with higher odds of decreasing and lower odds of increasing personal fertility ideals compared to maintaining stable fertility ideals. Women with more education had lower odds of increasing rather than maintaining their fertility ideals, but women who had more initial economic hardship had lower odds of decreasing than maintaining fertility ideals. Women who were employed part time or full time at Wave 1 had higher odds of decreasing than maintaining stable fertility intentions between waves, relative to women who were unemployed.

Three of the attitudes measures were also associated with changes in fertility ideals. Higher initial religiosity, initial fertility intentions, and initial importance of motherhood were associated with lower odds of decreasing compared to maintaining stable fertility ideals. Similarly, within-person increases in fertility intentions and importance of motherhood were associated with lower odds of decreasing rather than maintaining stable fertility ideals. We found that increases in importance of motherhood were associated with lower odds of increasing compared to maintaining stable fertility ideals.

Of the life course and social cues measures, only parity and age were associated with changes in fertility ideals. Women with one child at both waves had lower odds of increasing compared to maintaining stable fertility ideals, and women with three or more children at both waves had lower odds of decreasing fertility ideals compared to women with two children at both waves. Additionally, women who added a child had lower odds of decreasing their fertility ideals compared to women who had two children at both waves. Finally, older women had lower odds of decreasing than maintaining fertility ideals.

## 5. Conclusion

Among women of reproductive age in stable heterosexual relationships over about three years, most (69%) had stable fertility ideals. A substantial minority (31%) of women, however, changed fertility ideals, with equal proportions increasing or decreasing their personal ideal number of children. Therefore, personal fertility ideals do change over approximately three years for a substantial minority of women, and they increase for some women and decrease for other women. This pattern could not easily be discerned using OLS regression.

We outlined five expectations about the relationships between independent variables and changes in fertility ideals. First, we expected that women who have backgrounds associated with higher fertility rates (religiosity, friends with children) would have higher fertility ideals and that increases in religiosity or friends with children would be associated with increases in fertility ideals. Higher initial religiosity was associated with lower odds of decreasing fertility ideals, but, contrary to expectations, changes in religiosity were not associated with changes in fertility ideals. Also contrary to our first set of expectations, changes in friends with children were not associated with changes in fertility ideals. Therefore, we do not find support for the idea that fertility changes among friends shape fertility ideals. Initial religiosity is related to the stability of the personal ideal number of children. It is possible that three years is not a sufficient time frame to allow for changes in friends, family, and religiosity to occur or to shape fertility ideals.

Our second expectation was that women who perceive that parents or partners want children would have higher fertility ideals and would be less likely to decrease fertility ideals. In contrast to this second set of expectations, we did not find that the perception of parents or partners wanting the participant to have children was associated with changes in fertility ideals. This finding is not consistent with expectations of the social cognitive model of fertility intentions. This inconsistency may be because of the relatively short interval between waves (≤ 3 years) or because the times at which an individual is most susceptible to these social cues may come earlier in their lives. Future research would benefit from data that has measures of family of origin experiences (e.g., number of siblings, parental divorce) and that follows women over more time.

Our third set of expectations was that women with higher education, full time employment, and higher importance of leisure and work would be more likely to decrease their personal fertility ideals. We found partial support for our third set of expectations. Higher education was associated with lower likelihood of increasing fertility ideals. In addition, full-time employment at the initial interview was associated with higher likelihood of decreasing fertility ideals. Higher importance of leisure and higher importance of career, however, are not associated with decreases in fertility ideals. These findings indicate that actual time constraints, rather than perceived importance and priority of behaviors, may lead to downward adjustments (or lower likelihood of upward adjustment) of personal fertility ideals. Individuals seem to be reacting to the demands of their schedules. Their personal preferences regarding their career and leisure are more stable and were used to shape initial fertility ideals.

Our fourth set of expectations was that increases in parity or the importance of motherhood would be associated with higher likelihood of increases in fertility ideals. Consistent with the fourth set of expectations, parity and importance of motherhood are associated with changes in fertility ideals. Our expectations focused on changes in parity, but we found that women who had one child at both waves were less likely to increase fertility ideals and that women who stayed at three or more children were less likely to decrease fertility ideals. The addition of a child, but not of a first child, was associated with decreased odds of decreasing one’s fertility ideals.

We were unsure whether higher importance of motherhood would be associated with higher or lower fertility ideals because women with higher importance of motherhood might want more children, or else might want to invest more in fewer children. In this study, women with higher initial importance of motherhood were less likely to decrease fertility ideals than remain stable, and women who increased importance of motherhood were less likely to decrease or increase fertility ideals than to remain stable. This finding may be tapping into contradictory processes. First, women may experience an increase in their importance of motherhood but in turn decrease their personal fertility ideal because they wish to invest more resources into each child rather than have a higher number of children (Morgan and King 2001). Alternatively, women may increase their importance of motherhood and then yearn for more children to help satisfy this increase in their mothering desires.

Finally, we expected that increases in economic hardship would be associated with a decrease in fertility ideals. We did not find that changes in economic hardship were associated with changes in fertility ideals. This stability must be interpreted in terms of the mixed findings on how economic hardship and income are related to fertility (Dey and Wasoff 2010; Miller 1994). We did find, however, that women with higher initial economic hardship were less likely to decrease than to have stable fertility ideals. This stability may be related to other evidence showing that, in lower socioeconomic statuses, a sense of achievement and success may be derived from having children (Edin and Kefalas 2011).

As with all studies, this study has some limitations. First, we have looked at change over a relatively short time span only. We took advantage of existing data for this study; we have no rationale for determining the ideal time span over which to assess change in the personal ideal number of children. In addition, we had access to only two waves of data. It would be ideal to follow women from the beginning to the end of reproductive years to assess the degree of stability or revision in fertility ideals. Furthermore, some of our independent variables did not change much over a three-year period, thus making it impossible to determine whether change in these variables would be associated with changes in ideals. Another limitation is that we did not have access to such background characteristics as number of siblings, community norms, and changes in relationship status that previous research and theory suggest should be related to change in personal fertility ideals. Reporting a fertility ideal that is less than one’s current number of children could be seen as taboo, and therefore may be associated with some social desirability bias. Less than 3% of participants report a lower ideal than their current number of children. Much of the research on fertility ideals has focused on European countries; therefore, it is challenging to compare our findings to prior work. Nonetheless, we add insights to fertility ideals research from the US context. Future research will benefit from including men and/or conducting couple analyses to determine the relevance of gender for fertility ideals.

Even with these limitations, our findings that some women change personal fertility ideals, that personal fertility ideals are related to but unique from desires and intentions, and that several characteristics are associated with changes in fertility ideals reveal the value of this line of inquiry. Even though many people in the United States adopt the contemporary cultural two-child norm, the realities of raising children in a society with few supports for employed parents may curtail the realization of personal fertility ideals. Future research that includes younger women, that includes men, that measures partner influences on personal as opposed to general fertility ideals, and that compares people who do and do not meet their fertility ideals on psychosocial outcomes would be worthwhile. The findings will advance theories that attempt to explain fertility behavior and policies that are designed to ensure that (1) all children are born wanted and that (2) individuals and couples can meet fertility goals. Additionally, further work should be done internationally to better understand how this process may function differently based on varying normative parity climates. For countries such as Japan and Germany that have subreplacement fertility levels (Billari and Kohler 2004; Boling 2008), or sub-Saharan African countries that have fertility rates that far exceed replacement (Bongaarts and Casterline 2013), the malleability of an individual’s ideal number of children may present as an important point for intervention.

## Figures and Tables

**Table 1:** Descriptive statistics of 879 women in the analytical sample from the National Survey of Fertility Barriers

	Mean/p	St. Dev.	Min.	Max.
PFI wave 1	2.59	1.09	0	4
PFI wave 2	2.60	1.09	0	4
**Individual characteristics**				
Education	15.74	2.70	4	22
Employment status				
Unemployed	0.25			
Part-time	0.16			
Full-time	0.59			
Employment status Δ	−0.03	0.83	−2	2
Economic hardship	1.37	0.60	1	4
Economic hardship Δ	0.04	0.58	−2.5	2.5
**Attitudes**				
Importance of leisure	3.25	0.80	1	4
Importance of leisure Δ	−0.02	0.85	−3	3
Importance of career	3.20	0.85	1	4
Importance of career Δ	0.08	0.91	−3	3
Religiosity	−0.09	1.08	−3.21	1.65
Religiosity Δ	−0.00	0.47	−1.54	1.96
Intentions	−0.56	2.45	−3	3
Intentions Δ	−1.16	2.05	−6	6
Importance of motherhood	3.35	0.71	1	4
Importance of motherhood Δ	0.01	0.42	−1.6	1.58
**Life course and social cues**				
Importance to partner	3.23	0.84	1	4
Importance to partner Δ	−0.05	0.65	−3	3
Importance to parents	3.08	0.79	1	4
Importance to parents Δ	−0.00	0.71	−3	3
Friends have kids	3.98	0.76	1	5
Friends have kids Δ	0.05	0.78	−3	4
Always zero	0.18			
Always one	0.15			
Always two	0.24			
Always three plus	0.14			
Became a parent	0.10			
Added a child	0.19			
Age	35.35	5.79	25	45

*Note*: PFI = Personal fertility ideal.

**Table 2:** Multinomial logistic regression of change in personal fertility ideal compared to stable personal fertility ideal

	Decrease in PFI vs. stable		Increase in PFI vs. stable	
	RRR	CI	RRR	CI
Wave I PFI^1^				
Wave 1 PFI: 0 or 1	0.36	[0.09–1.42]	6.10 ***	[2.66–14.0]
Wave 1 PFI: 3 or more	17.51 ***	[8.74–35.1]	0.32 ***	[0.19–0.53]
**Individual characteristics**				
Education	0.96	[0.87–1.06]	0.82 ***	[0.75–0.89]
Employment status^2^				
Part-time	2.47 *	[1.19–5.12]	1.79	[0.90–3.57]
Full-time	2.24 *	[1.14–4.39]	1.55	[0.84–2.86]
Employment status Δ	1.27	[0.92–1.75]	0.94	[0.70–1.25]
Economic hardship	0.56 *	[0.35–0.90]	0.97	[0.66–1.41]
Economic hardship Δ	0.94	[0.61–1.46]	1.19	[0.82–1.73]
**Attitudes**				
Importance of leisure	0.67 *	[0.47–0.95]	1.23	[0.87–1.72]
Importance of leisure Δ	0.75	[0.54–1.04]	0.95	[0.71–1.28]
Importance of career	1.15	[0.82–1.61]	0.97	[0.72–1.31]
Importance of career Δ	1.20	[0.88–1.64]	0.93	[0.71–1.23]
Religiosity	0.74 **	[0.59–0.92]	0.91	[0.75–1.12]
Religiosity Δ	1.43	[0.89–2.32]	1.06	[0.69–1.62]
Intentions	0.72 ***	[0.60–0.87]	1.09	[0.93–1.28]
Intentions Δ	0.68 ***	[0.58–0.81]	1.04	[0.90–1.20]
Importance of motherhood	0.49 *	[0.25–0.97]	1.68	[0.94–3.02]
Importance of motherhood Δ	2.46 *	[1.19–5.08]	0.37 **	[0.19–0.72]
**Life course and social cues**				
Importance to partner	0.74	[0.45–1.23]	0.93	[0.61–1.43]
Importance to partner Δ	0.97	[0.62–1.53]	0.99	[0.64–1.52]
Importance to parents	0.86	[0.59–1.26]	1.26	[0.89–1.79]
Importance to parents Δ	1.16	[0.80–1.66]	1.22	[0.85–1.75]
Friends have kids	0.89	[0.59–1.33]	1.01	[0.71–1.42]
Friends have kids Δ	1.29	[0.88–1.87]	1.03	[0.76–1.41]
**Initial and Δ in parity**				
Always zero children	0.62	[0.23–1.70]	0.45	[0.19–1.11]
Always one child	1.65	[0.79–3.43]	0.42 *	[0.20–0.88]
Always three plus children	0.32 **	[0.15–0.67]	1.16	[0.56–2.42]
Became a parent	0.77	[0.27–2.20]	0.40	[0.15–1.05]
Added a child	0.27 ***	[0.12–0.58]	1.01	[0.47–2.16]
Age	0.88 ***	[0.83–0.93]	0.98	[0.93–1.03]
N	879			

*Notes:* PFI = Personal fertility ideal; RRR = Exponentiated coefficients; CI = 95% in brackets.

1 Wave I PFI of 2 is the reference category.

2 Unemployed is the reference category.

* p < 0.05,

** p < 0.01,

*** p < 0.001.
